# Education attainment of head of households associated with insecticide-treated net utilization among five to nineteen-year old individuals: evidence from the malaria indicator survey 2010 in Zambia

**DOI:** 10.1186/1475-2875-13-378

**Published:** 2014-09-23

**Authors:** Mwamba Sichande, Charles Michelo, Hikabasa Halwindi, John Miller

**Affiliations:** Department of Public Health, School of Medicine, University of Zambia, P.O Box 50110, Lusaka, Zambia; South Luangwa conservation Society, P.O Box 3, Mfuwe, Zambia; PATH Malaria Control and Evaluation Partnership in Africa, Postnet, P.O Box 370, P/Bag E-10, Lusaka, Zambia

**Keywords:** Malaria, Insecticide-Treated Nets (ITNs), Malaria Indicator Survey (MIS)

## Abstract

**Background:**

Education attainment may be a factor potentially influencing health-seeking behaviour of individuals. The effect of the level of education attainment of head of households of five to nineteen year old individuals in Zambia on ITN utilization was investigated.

**Methods:**

Data stem from the 2010 Malaria Indicator Survey, which covered the entire Zambia, was used in this study. Of the total number of five to 19-year olds (n = 7,429), only 65% (4, 810) met the inclusion criteria for this study. The education level of the head of households was taken as a household variable and was categorized as "never been to school" for those who had never enrolled in school, Primary for Grades 1 to 7, Secondary for Grades 8 to 12 and Tertiary for beyond Grade 12. Multivariate Logistic regression was used to determine adjusted odds ratios that estimated the effect of education on ITN utilization after controlling for residence, sex, age group and other background factors.

**Results:**

Overall (n = 4,810), 48.5% were males and 51.5% were females with the median age of 10 years and 11 years respectively. The ITN utilization among the five to 19 year old individuals from households with the head having Primary and Secondary education were not statistically significant from those who came from households where the head had never been to school. However, those who came from households with the head having tertiary education attainment were 1.7 times more likely to have slept under an ITN a night before the survey than those from households headed by individuals who never attended school or had primary education. (AOR, 1.69; 95% CI, 1.19-2.41). Of the eligible population, 35% were excluded from the study due to incomplete records.

**Conclusion:**

The findings suggest that tertiary education of the head of head of the household might be important in influencing health behaviour of the members of households. Therefore, health education messages focussing on strategies that aim to increase ITN utilization need to account for these differential variations associated with education attainment in communities.

## Background

Insecticide-treated nets (ITNs) have been found to reduce all forms of child mortality by 16% in most African settings making it an effective intervention for the control of malaria transmission by Anopheles mosquitoes [1]. However, high coverage and utilization rates are required for ITNs to make a substantial impact on the prevention of malaria transmission. In order to achieve this, the Government of the Republic of Zambia, in collaboration with cooperating partners embarked on distribution of free ITNs throughout the country. This led to an increase in the number of households owning at least one ITN from 13.6% in 2001 to 35% in 2005 and 64% in 2010 [2]. The current national policy on ITNs is to have all sleeping spaces available in the households covered by ITNs in order to increase the levels of utilization. This is a shift from making ITNs only available to pregnant women and under-five children to all at risk of malaria.

The Ministry of Health had, therefore, set a target to have 80% of the households owning at least one ITN and to increase the level of utilization to 75% by 2012 [3]. Although there has been an increase in the ITN coverage rates over the years, the ITN utilization trend by age group shows that five to 19-year old individuals are least likely to sleep under an ITN [4, 5] despite making up about 40% of the Zambian population [6]. This implies that the larger number of people at risk of malaria still remain unprotected by ITNs. Articles that have been published on the determinants of ITN utilization have focused more on pregnant women and under-five children [7, 8].

This study was aimed to identify if education attainment among household heads is associated with ITN utilization among five to 19-year olds in Zambia. The higher goal was to inform programming and associated intervention strategies.

## Methods

### Design

Data stem from the 2010 Malaria Indicator Survey (MIS) conducted in Zambia as part of national malaria surveillance programme. The MIS was a nationally representative cross-sectional survey that was conducted in April and May of 2010 in Zambia using a two cluster sampling approach. The objective of the survey was to monitor and evaluate the coverage and use of malaria intervention programmes that were being implemented such as ITNs, Indoor Residue Spraying (IRS), anti-malarial drugs and estimation of the prevalence of fever, malaria parasitaemia and anaemia.

The country was stratified into rural, urban and new IRS strata. The first stage involved selection of clusters using probability proportional to size and the second stage involved selection of households within selected clusters using systematic sampling method. A representative sample of 4,500 households was selected for the whole country. The full details of the survey have been described elsewhere [5].

### Data collection

Household and women questionnaires were used to collect socio-demographic as well as utilization data. The household questionnaire was administered to the head of each of the selected households and it was used to list all the members of the household and valuable goods in the household. This helped in identifying women aged 15 to 49 years who answered the women questionnaire. The head of the household answered questions on behalf of all household members including those relating to ITN ownership and usage among all household members. The women questionnaire was used to collect information from women aged 15 to 49 years about general malaria knowledge and also information related to access to information about malaria.

### Data analysis

Data was analysed using Stata® Version 11 (Stata Corporation, College Station, Texas). The analysis was restricted to five to nineteen year old individuals who came from households that had at least one ITN and a woman who was interviewed. The goal was to find out the proportion of individuals aged five to nineteen years who slept under an ITN a night before the survey. This information was given by the head of the household and was available for all the household members. The outcome factor was examined against its association with various personal and household factors. Personal factors examined were sex, residence and region while household factors included education level of the head of household, wealth index, and number of ITNs, age of the head of the household, presence of under-five children and presence of under five children who slept under an ITN the night before the survey. Education attainment of the head of the household was measured using the number of formal school years the respondent spent in school. This was categorized into four as follows; Never been to school for those who had no formal education, Primary level from Grades one to seven, Secondary level from Grades eight to twelve and Tertiary level for above Grade Twelve.

The first step involved using bivariate cross tabulation analysis where each of the individual and or household characteristics were tested to what extent they were associated with ITN utilization and the mantel-Haenszel (chi-square) test for overall degree of association was used for this measure.

Multiple Logistic regression analyses were used to assess and estimate the specific changes in odds among all the included individual and household factors on ITN utilization. The distribution of age as a continuous variable conformed to normality as assessed by probability plots. Interactions were looked for using the likelihood ratio test. Model diagnostics were done using the Maximum Likelihood Estimation (MLE) and the Hosmer-Lemeshow goodness-of-fit. The variables in the model were age, sex, residence and region while household variables included education level of the head of household, wealth index, number of ITNs, age of the head of household, presence of under-five children and presence of under-five children who slept under an ITN the night before the survey.

### Ethics

Prior to the survey, the Research Ethics Committee of the University of Zambia reviewed and approved the protocol. Permission was sought from the Ministry of Health to use the MIS 2010 dataset for this study and ethical clearance was obtained from the Research Ethics Committee of the University of Zambia for the protocol to conduct secondary data analysis for MIS 2010. Written informed consent was obtained from parents or guardians for the publication of this report and any accompanying images.

## Results

### Participation and distribution

Overall (n = 4, 810), 48.5% were males. The majority (68.2%) of the participants came from rural areas while 31.8% came from urban areas (Table 1). Copperbelt province contributed the highest number of participants (18.8%) while North Western had the least (5.5%). The mean age was 10.9 years and standard deviation was 4.2 years. Non-participation (35%) was largely due to incomplete records.

**Table 1:** **Distribution of socio demographic characteristics in 2010 malaria indicator survey in Zambia**

Characteristic	Category	Proportion (percent) n = 4810
Sex	Male	48.5%
	Female	51.5%
Residence	Rural	31.8%
	Urban	68.2%
Region	Luapula	6.8%
	Central	10.9%
	Copperbelt	18.8%
	Eastern	16.5%
	Lusaka	10.2%
	North Western	5.5%
	Northern	10.8%
	Southern	12.5%
	Western	8.1%
Age group (yrs)	5-9	41.8%
	10-14	34.7%
	15-19	23.5%
Gender of head of household	Male	79.6%
	Female	20.4%
Wealth quintile	Lowest	20.1%
	Second	12.5%
	Middle	19.5%
	Fourth	23.1%
	Highest	24.8%
Ratio of nets to sleeping spaces	Not all spaces covered	54.5%
	All spaces covered	45.5%
Education level of head of household	Never attended school	7.5%
	Primary	41.3%
	Secondary	39.4%
	Tertiary	11.8%
Age of head household (yrs)	<25	3.4%
	25-34	22.0%
	35-44	36.5%
	45-59	29.9%
	60+	8.2%
Number of ITNs	1	34.0%
	2	32.2%
	3 or more	33.8%

### Women knowledge on malaria

The majority (69.9%) came from households where the women knew that sleeping under an ITN protects against malaria. Of the study participants, 80.5% came from households where women knew that malaria parasite is transmitted by mosquitoes. The higher number of the participants (95.6%) had reported not having received health education about malaria at home.

The bivariate analysis showed that education level of the head of the household was associated with ITN utilization among five to19-year olds (p = 0.013) as shown in Table 2.

**Table 2:** **Distribution of number of 5–19 year olds who slept under ITN in households with at least one ITN**

Characteristic		Slept under ITN last night		Significance (p-value) using chi square test
		Yes n(%)	No n(%)	
Sex	Male	928(39.8)	1 404(60.2)	0.001
	Female	1 095(44.2)	1 383(55.8)	
Residence	Rural	552(36.1)	978(63.9)	0.005
	Urban	1 471(44.8)	1 809(55.2)	
Region	Luapula	91(28.0)	234(72.0)	0.000
	Central	236(44.9)	289(55.1)	
	Copperbelt	336(37.1)	570(62.9)	
	Eastern	429(54.1)	364(45.9)	
	Lusaka	151(30.9)	338(69.1)	
	North Western	136(51.7)	127(48.3)	
	Northern	252(48.3)	270(51.7)	
	Southern	207(34.7)	390(65.3)	
	Western	185(47.4)	205(52.6)	
Age group (yrs)	5-9	932(46.3)	1 080(53.7)	0.000
	10-14	647(38.8)	1 022(61.2)	
	15-19	444(39.3)	685(60.7)	
Number of 5–19 headed by Male or Female	Male	1 618(42.2)	2 213(57.8)	0.934
	Female	405(41.4)	574(58.6)	
Wealth quintile	Lowest	391(40.4)	576(59.6)	0.465
	Second	293(48.8)	307(51.2)	
	Middle	407(43.4)	531(56.6)	
	Fourth	450(40.4)	663(59.6)	
	Highest	482(40.4)	710(59.6)	
Ratio of nets to sleeping spaces	Inadequate nets	653(24.9)	1 969(75.1)	0.000
	Adequate nets	1 370(62.6)	818(37.4)	
Education level of head of household	Never attended school	150(41.8)	209(58.2)	0.013
	Primary	819(41.2)	1 168(58.8)	
	Secondary	764(40.3)	1 130(59.7)	
	Tertiary	290(50.9)	280(49.1)	
Age group household head (years)	<25	95(57.6)	70(42.4)	0.001
	25-34	508(48.0)	551(52.0)	
	35-44	734(41.8)	1 020(58.2)	
	45-59	514(35.8)	922(64.2)	
	60+	172(43.4)	224(56.6)	
Number of ITNs	1	268(16.4)	1 366(83.6)	0.000
	2	773(50.0)	774(50.0)	
	3 or more	982(60.3)	647(39.7)	

Multivariate logistic regression analysis was used to determine the effect of level of education of the head of the household on ITN utilization among five to 19-year olds from households with at least one ITN putting into account effect of survey settings (Table 3). The model was controlled for covariates that were significant at (p < 0.05) in the bivariate analysis. These covariates were sex, residence, region, age group of individuals, age group of the head of household, number of households with under-fives who slept under ITNs, number of ITNs and ratio of ITNs to sleeping spaces in households. There was no significant difference in ITN utilization between individuals who came from the household where the head of the households had secondary education (AOR = 1.15; 95% CI 0.89-1.49) and those from households where the head of households had primary level or had never been to school. However, individuals from households with tertiary education attainment for the head of the household were 1.7 times more likely to have slept under an ITN than those from households were the head had primary or never been to school (OR = 1.69; 95% CI 1.19-2.41).

**Table 3:** **Logistic regression of predictors of 5–19 year old sleeping under an ITN a night before the survey in households with at least one ITN**

Characteristic		AOR (95% CI)
Sex	Male	1.00
	Female	1.36(1.17-1.58)
Number of ITNs	1	1.00
	2	3.94(3.02-5.13)
	3 or more	5.11(3.63-7.20)
Region	Luapula	1.00
	Central	1.54(1.01-2.33)
	Copperbelt	1.22(0.77-1.93)
	Eastern	2.59(1.68-3.99)
	Lusaka	1.19(0.68-2.07)
	North Western	1.86(1.19-2.92)
	Northern	1.48(0.91-2.40)
	Southern	0.82(0.48-1.38)
	Western	1.69(1.09-2.61)
Residence	Rural	1.00
	Urban	1.20(0.85-1.68)
Education level of head of household	Primary and Never	1.00
	Secondary	1.15(0.89-1.49)
	Tertiary	1.69(1.19-2.41)
Number of bed spaces covered	Not all	1.00
	All	2.78(2.17-3.57)
Household with under five who slept under ITNs	No	1.00
	Yes	2.61(2.00-3.41)
Age group of head of households (years)	<25	3.18(1.77-5.69)
	25-34	1.37(1.03-1.82)
	35-44	1.00
	45-59	1.04(0.77-1.40)
	60 and above	1.34(0.82-2.18)
Age group of 5–19 year olds	5-9	1.29(1.03-1.60)
	10-14	0.92(0.76-1.13)
	15-19	1.00

Notes: 1. AOR denotes adjusted Odds Ratio 2. CI denotes confidence Interval.

## Discussion

This study finds evidence suggesting that education attainment is probably one of the important factors that could influence ITN utilization. The finding that tertiary level of education of the head of household is associated with high ITN utilization among five to 19 year olds suggests priority when planning health outreach programmes aimed at sensitizing people on ITN use should be focussed more on those with lower education. This trend may be due to the fact that the five to 19-year old individuals mostly depend on their parents or guardians to make decisions on their behalf and hence parents become important in determining whether their household members utilize ITNs or not. The parents’ knowledge about the danger of malaria to their children determines whether they take action or not to compel their children to use preventive measures such as sleeping under an ITN. This theory is based on health belief model [9] in health promotion. This was also demonstrated in a study which was done in Ethiopia, where skill based training of heads of households on ITN utilization increased ITN utilization in under five children by 31.6 per cent and 38.4 per cent after six and twelve months respectively [10].

Although receiving malaria education was significantly found to be associated with ITN utilization in women in Ethiopia [11], receiving malaria education at home did not result in increased ITN use by the five to 19-year olds in 2010 in Zambia. This may have been due to low coverage of health education at homes in 2010 since only 4.4 per cent of the 4, 810 participants had received health education on malaria at home. An increase in the coverage of health education at homes could have probably resulted in an increased ITN utilization by the 5 to 19 year olds as it would have bridged the gap that existed between heads of households with low and high levels of education. The number of ITNs owned by the household was also an important determinant of ITN utilization by the five to 19-year olds. Individuals who came from households with two ITNs were four times more likely to have slept under an ITN than those from households with one ITN. Similarly, individuals aged five to 19 years who came from households which had three or more ITNs were five times more likely to have slept under an ITN than those from the household which had only one ITN. This suggests that an increase in the number of households with at least three ITNs could lead to an increased ITN utilization among the five to 19-year olds.

This study has highlighted the importance of head of household education level in determining ITN utilization by household members and this can be useful in designing outreach programmes and targeting heads of households with low education levels could lead to increased ITN utilisation in five to 19-year olds.

There are some limitations to this study, the questionnaire was not specifically designed for this study and therefore, some important questions that could have helped in identifying other determinants were left out. There was no question on the number of five to 19-year olds who were sleeping on the floor although this has been found to be associated with low ITN utilization [12]. This would have been captured if mixed methods were used. The fact that this was a household based survey means that those individuals who were in boarding schools and colleges could not have been captured.

## Conclusion

The findings that tertiary education level of the head of households influenced the ITN utilization of the five to 19-year old individuals in the 2010 Malaria Indicator survey suggests that health education aimed at sensitizing the public on the importance of sleeping under an ITN as a preventive tool against malaria should focus more on targeting those with lower levels of education. This will help bridge the gap that exist in knowledge levels between those with higher and lower education attainment and might lead to an increase in ITN utilization in the five to19-year old age group.

Future research should focus on school-based surveys to target children using mixed methods and also to educate children on the importance of using ITNs as a preventative method against malaria. The authorities should also consider distributing ITNs in schools to enable more children access ITNs.

## Abbreviations

AOR: Adjusted odds ratio
OR: Odds ratio
ITN: Insecticide-treated net
IRS: Indoor residue spraying
CI: Confidence interval
MIS: Malaria indicator survey
WHO: World health organization.
